# LncRNA HOTAIR induces sunitinib resistance in renal cancer by acting as a competing endogenous RNA to regulate autophagy of renal cells

**DOI:** 10.1186/s12935-020-01419-0

**Published:** 2020-07-24

**Authors:** Dechao Li, Changfu Li, Yongsheng Chen, Lichen Teng, Yan Cao, Wentao Wang, Hongxin Pan, Yongpeng Xu, Dan Yang

**Affiliations:** 1 Department of Urological Surgery, Harbin Medical University Cancer Hospital, Harbin, 150086 Heilongjiang People’s Republic of China; 2 Department of Biochemistry and Molecular Biology, Harbin Medical University, No. 157, Baojian Road, Nangang District, Harbin, 150081 Heilongjiang People’s Republic of China

**Keywords:** HOTAIR, Sunitinib, miR-17-5p, Beclin1, Renal cancer, Autophagy, Drug resistance, LncRNA

## Abstract

**Background:**

Cell autophagy has been proposed to be involved in drug resistance therapy. However, how the long non-coding RNA (lncRNA) reduces risks of drug resistance in renal cancer (RC) cells needs a thorough inquiry. This study was assigned to probe the effect and mechanism of HOTAIR on sunitinib resistance of RC.

**Methods:**

Clinical RC tissues and para-carcinoma tissues were obtained to detect the expressions of miR-17-5p, HOTAIR and Beclin1. Sunitinib-resistant cells (786-O-R and ACHN-R) were constructed using parental RC cells (786-O and ACHN). The resistance of 786-O-R and ACHN-R cells to sunitinib was examined. Western blot and qRT-PCR were assayed to obtain the expressions of miR-17-5p, HOTAIR and Beclin1. The effects of HOTAIR knockdown or miR-17-5p overexpression/knockdown on cell autophagy and sunitinib resistance were measured by MDC staining, immunofluorescence and Western blot. The sensitivity of RC cells to sunitinib and change in cell clone formation after sunitinib treatment were assessed by CCK-8 assay and colony formation assay, respectively. The relationships among HOTAIR, miR-17-5p and Beclin1 were verified by dual-luciferase reporter gene and RIP assay. The role of HOTAIR knockdown in sunitinib resistance was verified in nude mice.

**Results:**

HOTAIR expression in sunitinib-resistant cells is higher than that in parental cells. Knockdown of HOTAIR in sunitinib-resistant cells lead to refrained sunitinib resistance and cell autophagy both in vivo and in vitro. Activation of autophagy could raise resistance to sunitinib in RC cells, while inhibition of autophagy could improve the sensitivity of sunitinib-resistant cells to sunitinib. HOTAIR could compete with miR-17-5p to regulate Beclin1 expression. Knockdown of miR-17-5p in parental cells increases cell resistant to sunitinib, and overexpression of miR-17-5p in sunitinib-resistant cells increases cell sensitive to sunitinib.

**Conclusion:**

HOTAIR negatively targets miR-17-5p to activate Beclin1-mediated cell autophagy, thereby enhancing sunitinib resistance in RC cells.

## Background

Renal cancer (RC), a highly vascularized neoplasm, is commonly connected with the mutations in the von Hippel-Lindau gene that promotes angiogenesis pathway [1]. The five-year survival rate for patients with metastatic RC is about 30%, whereas less than 10% of patients had a survival period longer than 5 years [2]. Sunitinib treatment has been proven to lengthen progression-free survival and overall survival in RC patients, but a large number of patients developed resistant to sunitinib, eventually resulting in cancer recurrence [1, 3]. Based on preclinical studies, several different mechanisms of resistance to sunitinib and other antiangiogenic tyrosine kinase inhibitors have been proposed, including induction of epithelial to mesenchymal transformation and alternative growth factor signaling, but failed to fully explain the clinical observations of RC resistance [4]. Therefore, an improved understanding of the potential mechanism on sunitinib resistance in RC is also necessary.

Autophagy is an extremely conservative metabolic process in eukaryotic cells that maintains the viability of cells under stable or stressed conditions [5]. Autophagy regulation has been reported to reverse the resistance to chemotherapy [6]. Additionally, the resistance and cytotoxicity of many chemotherapeutics are considered to be closely related to autophagy regulation. [7]. Inhibition of autophagic flux and sequestration in lysosomes were proved to result in resistance to sunitinib in renal cell carcinoma [8]. However, much still remains to be elucidated for autophagy regulation on sunitinib chemosensitivity in RC cells.

To date, long non-coding RNAs (lncRNAs) have a wide range of functions in chromatin modification and transcriptional, post-transcriptional and translational regulation [9]. Notably, lncRNAs were extensively involved in the germination and progression stage of diversified diseases including cell differentiation, cell cycle control, transcription, and translation [10, 11]. For example, energy stress-induced lncRNA FILNC1 could suppress c-Myc-mediated energy metabolism and RC development [12]. HOX transcript antisense intergenic RNA (HOTAIR) could serve as a biomarker to predict metastasis and poor prognosis in multiple tumors and act as a competing endogenous RNA (ceRNA) to up-regulate microRNA-204 to suppress invasion and migration of oesophageal cancer cells [13, 14]. Furthermore, in human cervical cancer, overexpression of HOTAIR may be deemed as a promising biomarker for predicting prognosis and recurrence [15]. Nevertheless, the role of HOTAIR in the development of sunitinib resistance in RC cells is still vague.

Here, we performed this research to inspect the possible mechanism of HOTAIR on cell autophagy and sunitinib resistance in RC cells. In this study, we obtained that knockdown of lncRNA HOTAIR improves the sensitivity of RC cells to sunitinib through inhibiting cell autophagy through mediating miR-17-5p/Beclin1 axis. This novel molecular mechanism for sunitinib resistance may utilize as a promising strategy for restore sunitinib sensitivity for RC patients.

## Materials and methods

### Ethical statement

The experimental scheme and study protocol concerning human and mouse were authorized by the Committee of Experimental Animals of Harbin Medical University. All procedures were in compliance with the Guide for the Care and Use of Laboratory Animals. All the patients provided their written informed consent.

### Tissue samples

A total of 33 RC tissues or para-carcinoma tissues were obtained from RC patients who underwent surgical removal. All samples were quickly preserved in liquid nitrogen for subsequent detection of HOTAIR, miR-17-5p and Beclin1 expressions.

### Cell culture

RC cell lines (786-O and ACHN) were purchased from the American Type Culture Collection (ATCC, Manassas, Virginia, USA). The 786-O and ACHN cells were incubated in RPMI-1640 medium (Gibco, Grand Island, NY, USA) containing 10% fetal bovine serum and 1% penicillin/streptomycin at 37 °C in a 5% CO_2_.

### Construction of sunitinib-resistant cell lines

The sunitinib resistance of RC cells was prepared according to method mentioned in a previous study [16]. Cells (5 × 10^6^) in the logarithmic growth phase were injected into nude mice by subcutaneous inoculation to establish subcutaneous model. After xenograft tumor size ≥ 200 mm^3^, nude mice were received 40 mg/kg normal saline or sunitinib by gavage once a day for 4 weeks. Mice were accordingly divided into the normal saline group and the sunitinib group. RC cells were isolated from tissues of mice in the sunitinib group after drug withdrawal for 2 weeks. The primary tumor cells were subcutaneously inoculated into nude mice for obtaining the second passaged RC cells. With reference to the above steps, tumor cells of the third generation were acquired, cultured and passaged in vitro. The sunitinib-resistant RC cell lines (786-O-R and ACHN-R cells) were successfully established if no difference on tumor size was noticed between the normal saline group and the sunitinib group.

### Establishment of RC xenograft model in nude mice

Four to six-week-old male BALB/c nude mice (purchased from Guangdong Medical Laboratory Animal Center) were randomly divided into four groups (n = 8 in each group) and housed in specific pathogen free conditions. All procedures were in compliance with Regulations for the Administration of Affairs Concerning Experimental Animals. After anesthesia, the nude mice placed in a right lateral position. A 2 cm incision was made in the left dorsal skin and peritoneum under pathogen-free circumstance. The kidney was gently squeezed out and fixed with moistened sterile gauze. The renal capsule was gently clamped with an ophthalmic forceps and injected with 1 × 10^6^ luciferase-labeled 776-O-R and ACHN-R cells. After that, the incision was sutured. The fluorescence signal intensity in the renal region of nude mice was measured weekly by in vivo optical imaging system (IVIS Spectrum, Caliper, USA) [16]. Tumor diameters were checked once a week exploiting calipers (tumor size = (length × width^2^)/2). When the tumor size reached 200 mm^3^, mice were grouped into suni + sh-NC group (tail vein injection of 50 nM sh-NC twice a week) and suni + sh-HOTAIR group (tail vein injection of 50 nM sh-HOTAIR twice a week) in the third week. All mice were administered with 40 mg/kg sunitinib once a day by gavage from the third week. In the seventh week, the expressions of HOTAIR, miR-17-5p and Beclin1 in the xenograft tissues were measured. Western blot was applied to evaluate the expressions of LC3-II/LC3-I and p62. Ki67 expression was detected by immunohistochemistry.

### Immunohistochemistry

Paraffin sections (4 μm) were prepared by fixing the tumor tissues in 4% paraformaldehyde for 48 h. After baking in an oven for 30 min, paraffin sections were stained by immunohistochemistry. Paraffin sections were deparaffinized with xylene, followed by distilled water wash. After phosphate-buffered saline (PBS) washing for 1 min, paraffin sections were incubated with the rabbit monoclonal antibody of Ki67 (ab16667, 1:200, Cambridge, MA, USA) at 4 °C overnight. After PBS washing three times, sections were then incubated with the secondary antibody for 1 h at room temperature and stained with diaminobenzidine for 1–3 min. Paraffin sections were washed three times with PBS to terminate color development. Nuclei were counterstained with hematoxylin, and then paraffin sections were dehydrated, permeabilized, and sealed. The positive expression of Ki67 was mainly located in cell nuclei, as evidenced by the presence of yellow or brown-yellow granules. The percentage of positive cells was counted.

### Cell transfection

sh-HOTAIR, sh-NC, miR-17-5p mimic, mimic-NC, miR-17-5p inhibitor, and inhibitor NC were purchase from Shanghai GenePharma Co., Ltd. (Shanghai, China). Transfection was accomplished with the lipofectamine 2000 transfection kit (Invitrogen, Carlsbad, CA, USA), according to the instructions. RC cells were transfected with above plasmid and accordingly grouped into 786-O-R + sh-HOTAIR group, ACHN-R + sh-HOTAIR group, 786-O + miR-17-5p inhibitor group, ACHN + miR-17-5p inhibitor group, 786-O-R + miR-17-5p mimic group, and ACHN-R + miR-17-5p mimic group.

### Quantitative reverse transcription polymerase chain reaction (qRT-PCR)

Total RNA was extracted from RC cells using TRIzol (Invitrogen, Carlsbad, CA, USA). Reverse transcription was performed with the reverse transcription kit (TaKaRa, Tokyo, Japan). All operations were performed following the manufacturer’s instructions. The expression of gene was inspected by LightCycler 480 qPCR instrument (Roche, Indianapolis, IN, USA) on conditions instructed on the fluorescence quantitative PCR kit (SYBR Green Mix, Roche Diagnostics, Indianapolis, IN). The reaction conditions were 95 °C for 10 s, followed by 45 cycles of 95 °C for 5 s, 60 °C for 10 s, and 72 °C for 10 s. A final extension was performed at 72 °C for 5 min. The test was done in triplicate. The internal references were presented as U6 or GAPDH, and data analysis employed 2^−ΔΔCt^ method [17]. The formula is as follows: ΔΔCt = [Ct_(target gene)_-Ct_(reference gene)_]_experimental group_-[Ct_(target gene)_-Ct_(reference gene)_] _control group_. The amplified primer sequences of each gene and its primers are listed in Table 1.

**Table 1:** Primer sequence information for reverse transcript polymerase chain reaction

Name of primer	Sequences
miR-17-5p-F	GATGGACGTGACATTCG
miR-17-5p-R	GCAGGGTCCGAGGTATTC
U6-F	CTCTCGCTTCGGCAGCACA
U6-R	ACGCTTCACGAATTTGCGT
HOTAIR-F	GGGGAAAATTTGGGAAGGCG
HOTAIR-R	TGCAAACCAATCGCCTGAAC
Beclin1-F	GTAGACCGGACTTGGGTGAC
Beclin1-R	TACCACATGCCTTGGTGACG
GAPDH-F	GCAAGGATGCTGGCGTAATG
GAPDH-R	TACGCGTAGGGGTTTGACAC

F, forward primer; R, reversed primer

### Western blot

Cells were lysed with RIPA lysate (Beyotime Biotechnology, Shanghai, China) to obtain protein samples. After the protein concentration was analyzed by BCA kit (Beyotime), the corresponding volume of protein was added to the loading buffer (Beyotime) and mixed. The proteins were placed in a boiling-water bath for 3 min of denaturation. Electrophoresis was conducted for 30 min at 80 V, and then for 1–2 h at 120 V once bromphenol blue reached the separation gel. Then proteins were transferred to membranes at 300 mA for 60 min in ice-bath. The membranes were rinsed 1–2 min with washing solution and then sealed in the blocking solution at room temperature for 60 min, or sealed overnight at 4 °C. After blocking, the PVDF membranes were incubated with the primary antibodies against GAPDH (5174S, 1: 1000), Beclin1 (3495S, 1: 1000), LC3-I/LC3-II (3868S, 1: 1000), and p62 (88588S, 1:1000) (Cell Signaling, Boston, USA) at room temperature in a shaking table for 1 h. Following primary incubation, the membranes were washed three times with washing solution for 30 min and then incubated with goat anti-rabbit IgG (1:5000, Beijing ComWin Biotech Co., Ltd., Beijing, China) labeled with horseradish peroxidase for 1 h at room temperature. After the membranes washed three times for 30 min, developing liquid was added to membranes for color development. Then chemiluminecence imaging analysis system was applied for observation.

### Monodansylcadaverine (MDC) staining

The parental cells (786-O and ACHN) and sunitinib resistance cells (786-O-R and ACHN-R) were trypsinized and then soaked in culture medium containing 10 μM of sunitinib or dimethylsulfoxide (DMSO) for 24 h. After that, culture medium was removed and cells were incubated with 50 μM of MDC in 37 °C for 60 min. PBS washing for three times before cells were fixed in 4% paraformaldehyde at 4 °C for 15 min. Then cells were subjected to observation of autophagic vacuoles under an inverted fluorescence microscope (Eclipse TE2000-U, Nikon, Tokyo, Japan). In addition, cell autophagy was activated after cell lines received 200 nM autophagy-activator rapamycin (RAP, Sigma-Aldrich, Merck KGaA, Darmstadt, Germany) for 12 h. Meanwhile, 5 mM autophagy inhibitor 3-methyladenine (3-MA, Sigma-Aldrich, Merck KGaA, Darmstadt, Germany) was applied to inhibit cell autophagy in 786-O-R and ACHN-R.

### Immunofluorescence staining

Cells were seeded on twenty-four well plates at the concentration of 5, 000 cells/well, fixed in 4% paraformaldehyde for 15 min, and permeated 20 min with 0.5% triton-X-100. Then sections were incubated with the primary antibody against LC3 (3868S, 1:200, Cell Signaling Technology, Danvers, MA, USA) overnight at 4 °C. Subsequently, sections were incubated with Alexa Fluor 488-labeled goat anti-rabbit IgG (ab150077, 1:2000, abcam, MA, USA) for 45 min avoiding light, followed by 3 × 5 min of PBS washing. Then, sections were stained with 0.5 µg/mL DAPI for 5 min in the dark prior to 2 × 5 min of PBS washing. Sections were sealed and then observed under a fluorescence microscope (BX61FL, OLYMPUS, Tokyo, Japan).

### CCK-8 assay

The dose–response of RC cells to sunitinib was determined by CCK-8. After digestion, cells were seeded on 96-well plates at a density of 3, 000 cells/well. Three wells were set for each group. Different concentrations (0, 1, 2, 5, 10 or 20 μM) of sunitibib were added to the medium for 24 h of cell incubation, after that, 10 µL of CCK-8 reagent (Tokyo, Dojindo, Japan) was added for 2 h of further incubation. Absorbance was inspected at 450 nm with a microplate absorbance reader.

### Colony formation assay

After trypsinization and centrifugation at 1500 rpm at 25 °C for 5 min, cells were re-suspended in complete medium. Subsequently, cells were cultured at a density of 500 cells/well in six-well plates containing 2 mL of 37 °C pre-warmed complete culture medium. Sunitinib was added to the sunitinib group and DMSO was added to the control group for cell culture at 37 °C with 5% CO_2_. Culture medium was removed in response to visible colonies. Cells were washed with PBS for twice and fixed in 1.5 mL of methanol for 15 min. Then the methanol was removed from the plate, and cells were stained with 1 mL of Giemsa staining solution avoiding light. About 20 min later, cells were washed with running water to rinse off the Giemsa staining solution. The 6-well plate was dried and cell clones were counted.

### Dual-luciferase reporter gene assay

The starBase (http://starbase.sysu.edu.cn/) was employed to predict the bind sites of HOTAIR and miR-17-5p as well as miR-17-5p and Beclin1. The mutated type sequences and wild type sequences in the binding sites of HOTAIR and miR-17-5p as well as miR-17-5p and Beclin1 were determined in accordance with the predicted results (wt-HOTAIR and mut-HOTAIR, and wt-Beclin1 and mut-Beclin1), and cloned into luciferase expression vector (pGL3-Basic). Then the vectors were co-transfected with 30 nM of miR-17-5p or miR-NC, respectively, into HEK293T cells. To avoid errors caused by of transfection efficiency, the ph-RL-TK plasmid was also transfected into cells as a control. Firefly luciferase activity and Renilla luciferase activity were detected by the dual-luciferase reporter gene assay system, and the luciferase activity was calculated as the ratio of Firefly luciferase activity and Renilla luciferase activity. The luciferase activity in the control group was set as 1 (100%). The internal reference was regarded as Renilla luciferase activity.

### RNA immunoprecipitation (RIP) Assay

Cells were washed with pre-cooled PBS for twice and centrifuged at 1500 rpm for 5 min followed by cell lysis with RIP lysate. The magnetic beads were re-suspended, and the cells were cultured with 5 μg of Ago2 antibody (2897S, 1:50, Cell Signaling, Boston, USA) or IgG antibody for 30 min at room temperature. The vortex oscillation of cells and 500 µL of RIP Wash Buffer was conducted thrice, and the mixture was placed on ice. The magnetic bead tube was placed on the magnetic rack, and the supernatant was removed. The 900 µL of RIP Immunoprecipitation Buffer was placed into each tube. Cell lysates were centrifuged at 14,000 rpm at 4 °C for 10 min, and 100 µL of supernatant was aspirated into the magnetic bead-antibody complex to make the total volume to 1 mL. Following incubation overnight at 4 °C, the complex was subjected to centrifugation and supernatant removal. Then the centrifugal tube was exposed to 500 µL of RIP Wash Buffer for vortex oscillation before cell supernatant was abandoned. The complex was washed with RIP Wash Buffer for 6 times. RNA purification: Each sample was re-suspended with 150 μL of Proteinase K Buffer magnetic bead-antibody complex and incubated at 55 °C for 30 min. Then the samples were placed on the magnetic rack to discard the supernatant. The expression of HOTAIR and Beclin1 was estimated by qRT-PCR after RNA extraction.

### Statistical analysis

Data were analyzed utilizing GraphPad prism7 software, and data were displayed as mean ± standard deviation (SD). T test was applied to the comparison between two groups. Comparisons among multiple groups were analyzed by one-way analysis of variance (ANOVA) and confirmed by Dunnett’s multiple comparisons test. The statistical significance was set at *P* < 0.05 and statistically highly significant as *P* < 0.001.

## Results

### Establishment of sunitinib-resistant cell lines

**Fig. 1:**
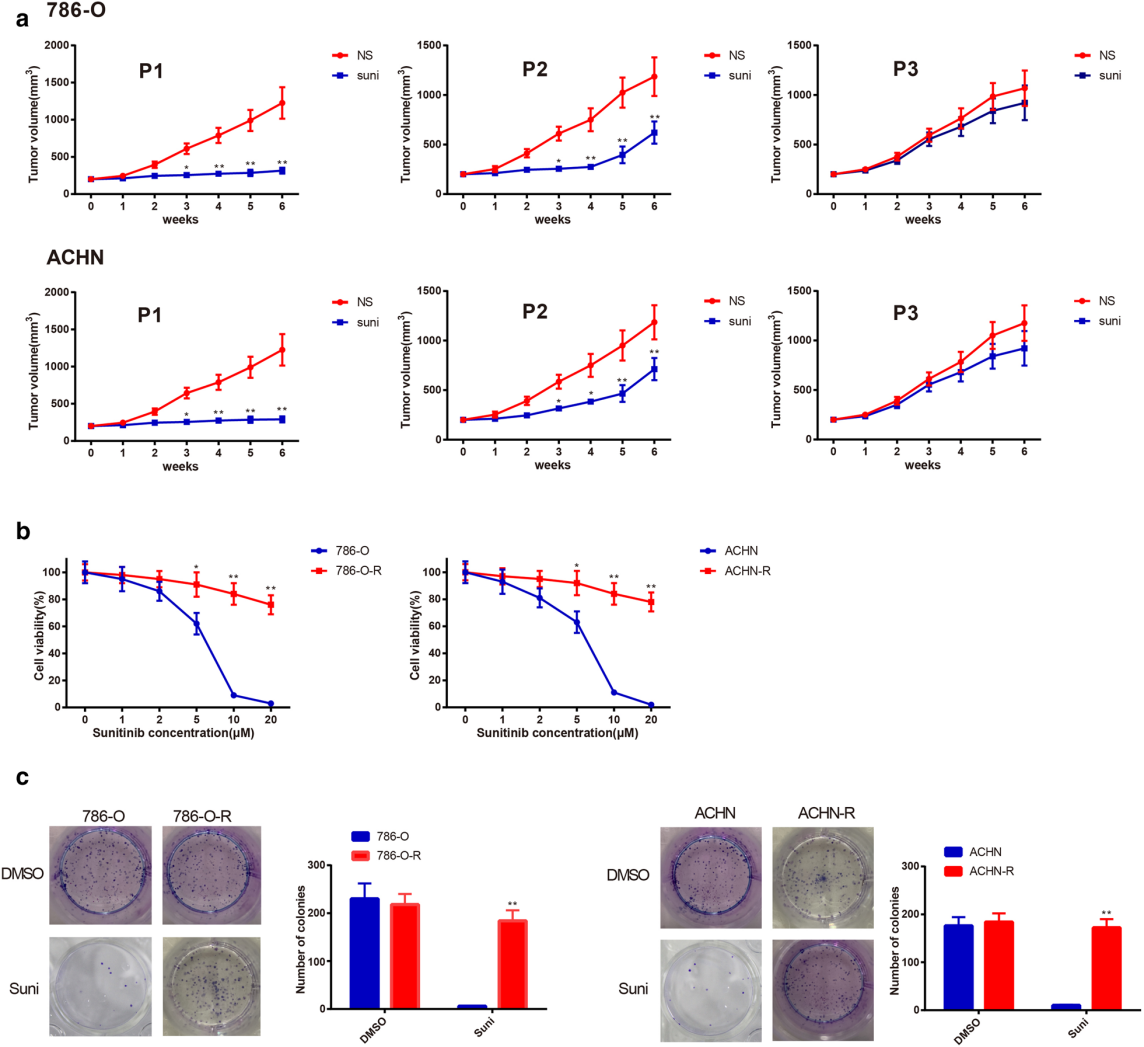
Establishment of cell line resistant to sunitinib. Tumor size was recorded after nude mice were injected with 786-O, ACHN, first-generation or second-generation tumor cells. The tumor cells in the third-generation were found to resistant to sunitinib (**a**). The reactions of parental cells (786-O and ACHN) and sunitinib-resistant cells (786-O-R and ACHN-R) to sunitinib were measured by CCK-8 assay. High concentration of sunitinib significantly inhibited the activity of 786-O and ACHN cells (**b**). Colony formation assay was employed to count the clone number of parental cells and sunitinib-resistant cells after 10 μM of sunitinib or DMSO treatment. Sunitinib at 10 μM markedly repressed the formation of parental cell clones, but had no effect on sunitinib-resistant cells (**c**). ^*^P < 0.05, ^**^P < 0.01, compared to NS or 786-O or ACHN group; DMSO, dimethylsulfoxide; NS, normal saline

After nude mice received passaged RC cells by subcutaneous injection, the tumor size of xenograft was calculated weekly. We found that sunitinib treatment had no effect on tumor size of mouse received third-generation xenograft (Fig. 1a), presenting the xenograft exhibited sunitinib resistance. Cells isolated from the third generation xenograft were renamed as 786-O-R and ACHN-R. CCK-8 and colony formation assay demonstrated that sunitinib had little influence on cell colony formation ability and cell viability in both 786-O-R and ACHN-R when compared with 786-O and ACHN cells (Fig. 1b, c). All those indicated that 786-O-R and ACHN-R resistant to sunitinib were successfully isolated.

### Knockdown of LncRNA HOTAIR inhibits autophagy and sunitinib resistance in RC cells

**Fig. 2:**
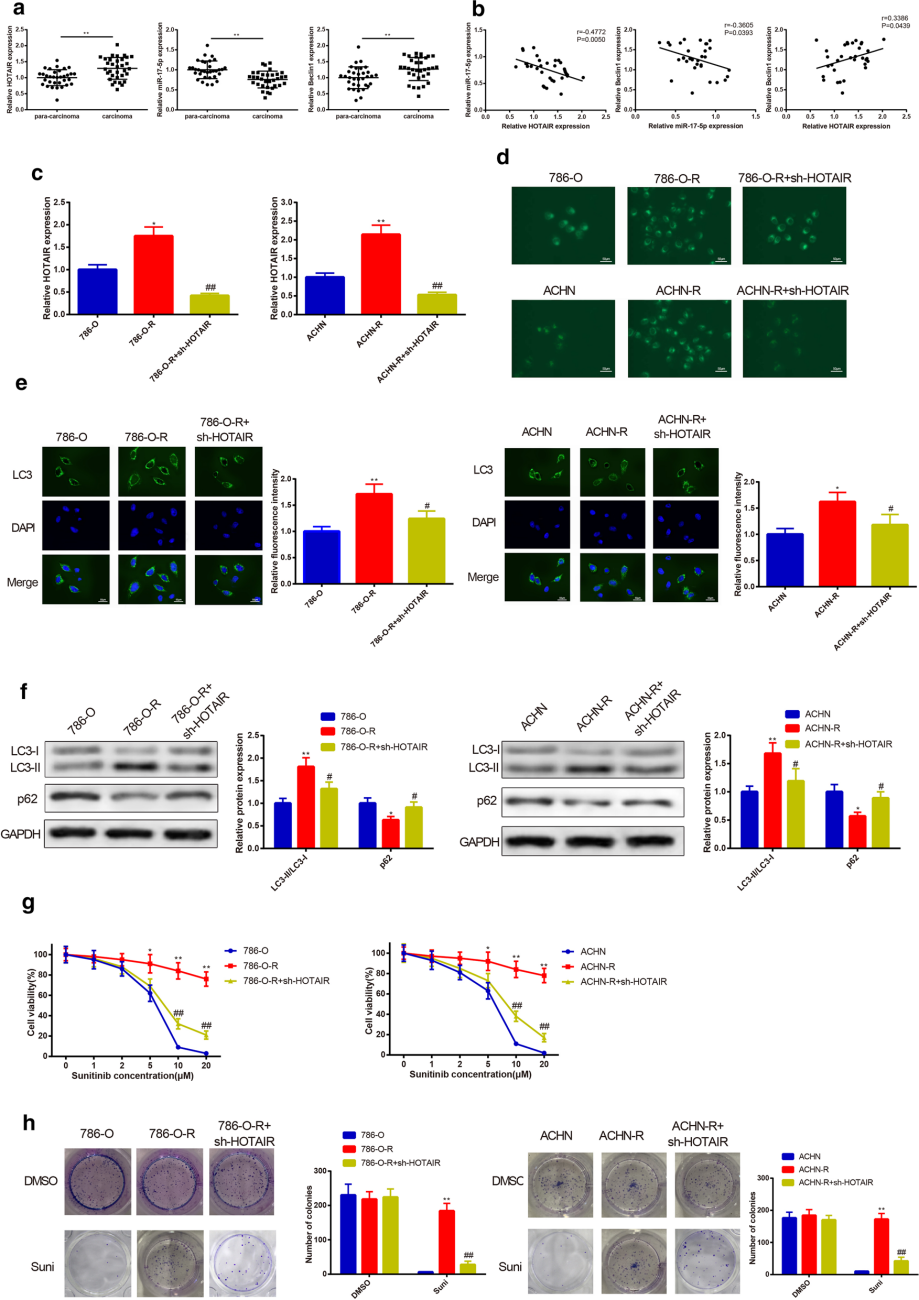
Effect of HOTAIR knockdown on autophagy and sunitinib resistance in 780-O-R and ACHN-R cells. Highly expressed HOTAIR and Beclin1 as well as lowly expressed miR-17-5p in RC tissues were inspected by qRT-PCR in comparison to those in para-carcinoma tissues (**a**). The negative correlation of miR-17-5p and HOTAIR or Beclin1, as well as positive correlation between HOTAIR and Beclin1 were detected (**b**). The parental and sunitinib-resistant cells were transfected with sh-HOTAIR. qRT-PCR accessed that transfection with sh-HOTAIR restrained the mRNA level of HOTAIR in RC cell lines (**c**). Then the effect of HOTAIR knockdown on cell autophagy was performed. Reduced autophagic vacuoles detected by MDC staining were found in RC cells which transfected with sh-HOTAIR (**d**). Immunofluorescence performed that expression intensity of LC3 was diminished after sh-HOTAIR transfection (**e**). The elevated protein expression of p62 and reduced LC3-II/LC3-I ratio in RC cells transfected with sh-HOTAIR were evaluated by Western blot (**f**). After cell transfection of sh-HOTAIR, sensitivity of RC cells to sunitinib was determined. The enhanced sensitivity of RC cells to sunitinib was measured by CCK-8 assay (**g**). After RC cells treated with 10 μM sunitinib or DMSO, the inhibited cell colony formation ability was assessed using colony formation assay (**h**). ^*^P < 0.05, ^**^P < 0.01, compared to 786-O or ACHN group; ^#^*P* < 0.05, ^##^*P* < 0.01, compared to 786-O-R or ACHN-R group; RC, renal cancer; DMSO, dimethylsulfoxide

The expressions of HOTAIR, miR-17-5p and Beclin1 in RC tissues and para-carcinoma tissues were analyzed by qRT-PCR. The results unfolded that highly expressed HOTAIR and Beclin1 and lowly expressed miR-17-5p were discovered in the RC tissues (Fig. 2a) (*P* < 0.05). Additionally, the level of miR-17-5p was negatively correlated with levels of HOTAIR and Beclin1, and HOTAIR expression was positively correlated with Beclin1 expression (Fig. 2b).

Detection on HOTAIR by qRT-PCR displayed higher expression of HOTAIR in both 780-O-R and ACHN-R cells rather than that in 780-O and ACHN cells, respectively (Fig. 2c) (*P* < 0.05). Transfection of sh-HOTAIR resulted in down-regulation of HOTAIR in both 780-O-R and ACHN-R cells (Fig. 2c) (*P* < 0.01).

Next, MDC staining, immunofluorescence and Western blot were employed to assay the effect of HOTAIR on cell autophagy of RC cells. 780-O-R and ACHN-R cells had more autophagic vacuoles than those in 780-O and ACHN cells, while following inhibition of HOTAIR shorted the autophagic vacuoles in 780-O-R and ACHN-R cells (Fig. 2d). The detection result on fluorescence intensity of LC3 was consistent with findings obtained from MDC staining that LC3 was highly expressed in both 780-O-R and ACHN-R cells and transfection of sh-HOTAIR plasmid reduced the expression of LC3 in 780-O-R and ACHN-R cells (Fig. 2e) (*P* < 0.05). To further confirm the inhibitory effect of HOTAIR suppression on autophagy of RC cells, we further analyzed expressions of autophagy-related proteins (LC3-II/LC3-I and p62) in RC cells before and after sh-HOTAIR transfection. The results revealed that 780-O-R and ACHN-R cells had elevated LC3-II/LC3-I ratio and decreased p62 expression, while transfection of sh-HOTAIR reversed these expression patterns in 780-O-R and ACHN-R cells (Fig. 2f) (*P* < 0.05).

To verify the possible role of HOTAIR on sunitinib sensitivity and viability of 780-O-R and ACHN-R cells, CCK-8 assay and colony formation assay were utilized to assess sunitinib sensitivity and cell viability. Result of CCK-8 implied that transfection of sh-HOTAIR enhanced sensitivity of 780-O-R and ACHN-R cells to sunitinib (Fig. 2g) (*P* < 0.05). In addition, inhibition of HOTAIR suppressed cell colony formation abilities of 780-O-R and ACHN-R cells (Fig. 2h) (*P* < 0.05). Collectively, cells resistant to sunitinib had elevated cell autophagy ability and sunitinib resistance in contrast to 780-O and ACHN cells, while knockdown of HOTAIR could decrease cell autophagy level and sunitinib resistance.

### Cell autophagy regulates sunitinib resistance of RC cells

**Fig. 3:**
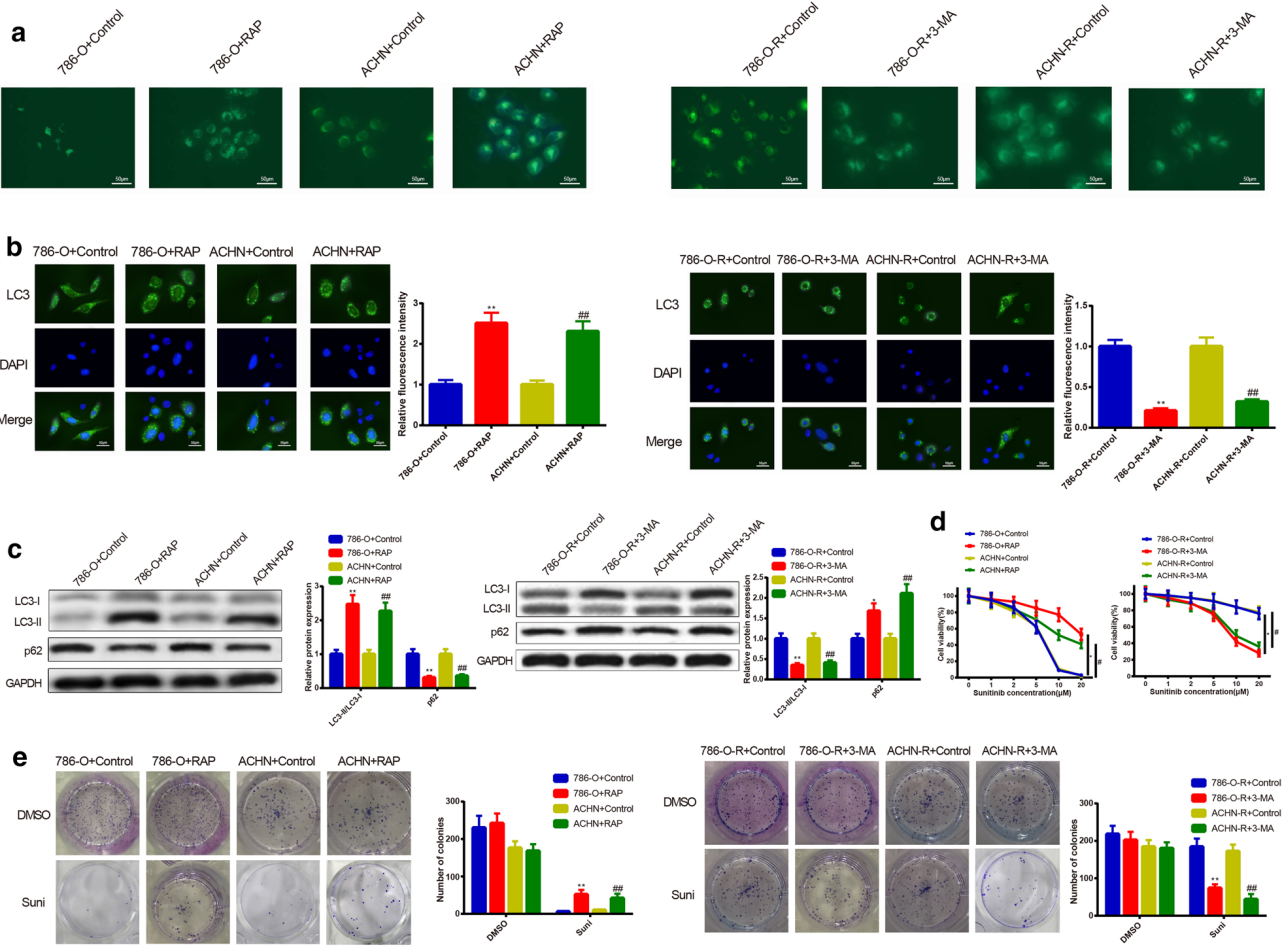
Effect of cell autophagy on sunitinib resistance in RC cells. To verify the regulatory role of cell autophagy in sunitinib resistance of RC cells, the parental cells (786-O and ACHN) were subjected to RAP for activation of cell autophagy. Meanwhile, sunitinib-resistant cells (786-O-R and ACHN-R) were exposed to 3-MA for inhibition of cell autophagy. Increased autophagic vacuoles of parental cells after RAP treatment as well as reduced autophagic vacuoles of sunitinib-resistant cells after 3-MA subjection were conducted by MDC staining (**a**). The enhanced fluorescence intensity of LC3 in RAP-induced group and suppressed fluorescence intensity of LC3 in the 3-MA-treated group were estimated by immunofluorescence (**b**). Protein expressions of LC3-II/LC3-I and p62 were assessed by Western blot. RAP potentiated LC3-II/LC3-I protein levels and lowered p62 expression in 786-O and ACHN cells. In 786-O-R and ACHN-R cells, 3-MA elevated protein expression of p62 and restrained LC3-II/LC3-I levels (**c**). CCK-8 assay was utilized to test the RC cell sensitivity to sunitinib. RAP was found to enhance the sunitinib resistance of parental cells, while 3-MA was discovered to potentiate the sunitinib sensitivity of sunitinib-resistant cells (**d**). Colony formation was applied to observe the formation of clone after exposure to 10 μM of sunitinib or DMSO. RAP-treated group had increased colony formation, while 3-MA-treated group possessed lowered colony formation (**e**). ^*^P < 0.05, ^**^P < 0.01, compared to 786-O + Control group or 786-O-R + Control group; ^#^*P* < 0.05, ^##^*P* < 0.01, compared to ACHN + Control or ACHN-R + Control group; RC, renal cancer; RAP, rapamycin; 3-MA, 3-methyladenine

We further investigate the relationship of cell autophagy and sunitinib resistance in RC cells. Firstly, 786-O and ACHN cells were subjected to 200 nM of RAP for 12 h, and then MDC, immunofluorescence and Western blot were applied to inspect cell autophagy ability. The results displayed that the 786-O + RAP group or ACHN + RAP group had more autophagic vacuoles (Fig. 3a), higher fluorescence intensity of LC3 (Fig. 3b) and LC3-II/LC3-I ratio, in addition to lower p62 expression in comparison to the 786-O + Control group or ACHN + Control group (Fig. 3c). Collectively, those data indicated that RAP activated autophagy of 786-O and ACHN cells.

The effect of cell autophagy on RC cells resistant to sunitinib was further explored. The detection on cell sensitivity to sunitinib manifested RAP treatment decreased cell sensitivity to sunitinib (Fig. 3d) (*P* < 0.05). Colony formation assay demonstrated that 786-O + RAP group or ACHN + RAP group had enhanced cell clone formation ability in comparison to the 786-O + Control group or ACHN + Control group (Fig. 3e) (*P* < 0.05). Collectively, those data indicated that cell autophagy could increase sunitinib resistance of 786-O and ACHN cells.

The aforementioned experiments were conducted in sunitinib-resistant cells for further verification. After 786-O-R or ACHN-R cells received 5 mM of 3-MA for 12 h, we found that inhibition on cell autophagy resulted in decreased autophagic vacuoles (Fig. 3a), fluorescence intensity of LC3 (Fig. 3b) and LC3-II/LC3-I ratio, as well as enhanced expression of p62 (Fig. 3c). Moreover, inhibition on cell autophagy also elevated cell sensitivity to sunitinib (Fig. 3d) and inhibited cell clone formation ability (Fig. 3e). Collectively, 3-MA could inhibit cell autophagy in 786-O-R and ACHN-R cells, which in turn could decrease the sunitinib resistance. Taken together, regulation on cell autophagy could alter the sunitinib resistance of RC cells.

### HOTAIR binds to miR-17-5p to regulate expression of Beclin1

**Fig. 4:**
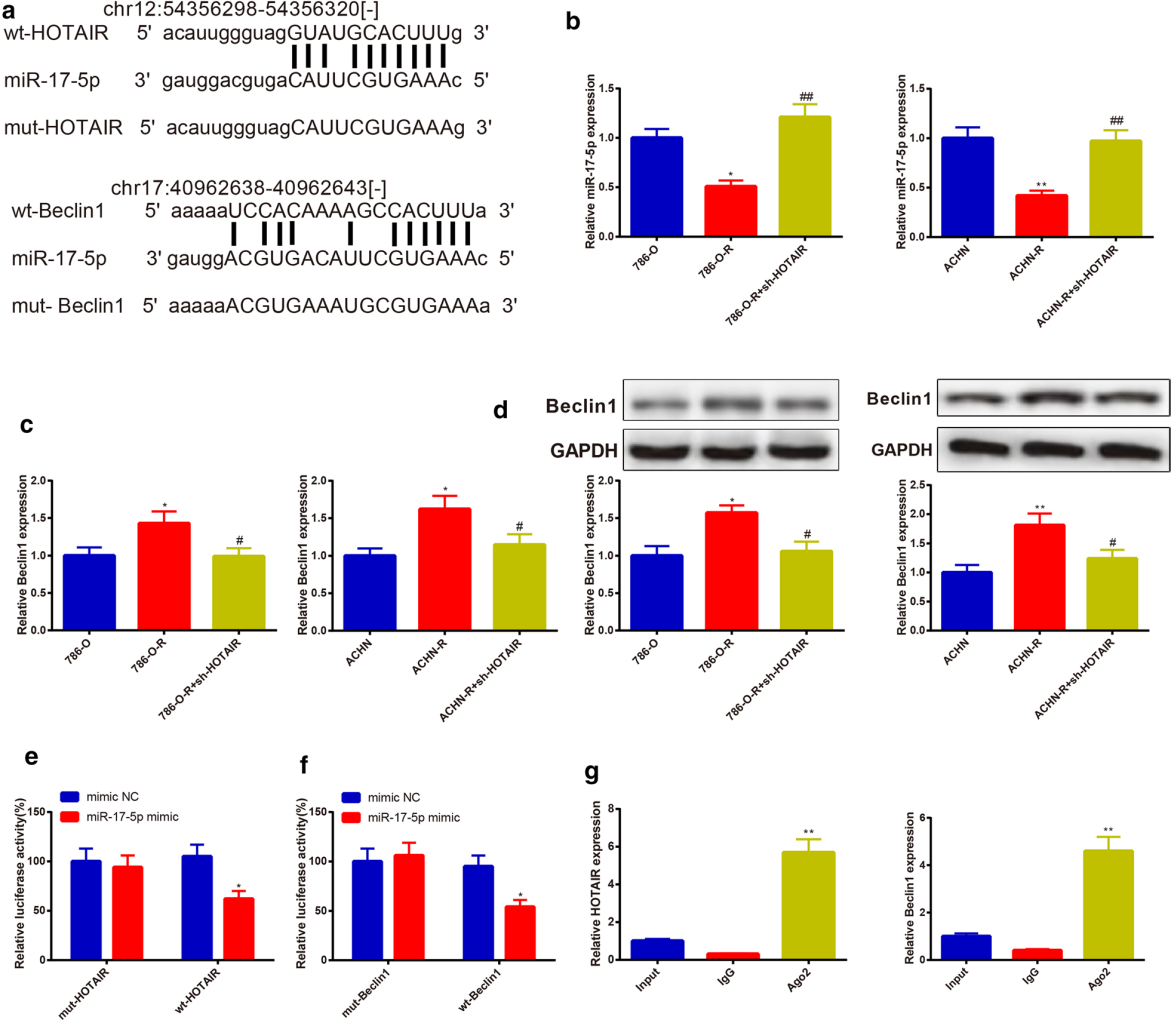
HOTAIR regulates the expression of Beclin1 via binding to miR-17-5p. The binding sites of miR-17-5p and HOTAIR or miR-17-5p and Beclin1 were predicted. Then the wild type sequences of HOTAIR and Beclin1, as well as the mutated type sequences of HOTAIR and Beclin1 were accordingly designed (**a**). After sh-HOTAIR was transfected into 786-O-R, or ACHN-R cells, the expressions of miR-17-5p and Beclin1 were assessed by qRT-PCR and Western blot. The expression of miR-17-5p in sunitinib-resistant cells was lower than that in parental cells. Transfection with sh-HOTAIR enhanced the mRNA expression of miR-17-5p (**b**). The sunitinib-resistant cells had higher mRNA and protein levels of Beclin1 than parental cells. Knockdown of HOTAIR suppressed Beclin1 (**c**, **d**). The dual-luciferase reporter gene and RIP assay were employed to inspect the binding sites of HOTAIR and miR-17-5p as well as miR-17-5p and Beclin1 (**e**–**g**). ^*^P < 0.05, ^**^P < 0.01, compared to 786-O or ACHN or mimic NC or IgG group; ^#^*P* < 0.05, ^##^*P* < 0.01, compared to 786-O-R or ACHN-R group; RIP, RNA immunoprecipitation

Starbase (http://starbase.sysu.edu.cn/agoClipRNA.php?source=mRNA) predicted the binding relationship of HOTAIR with miR-17-5p, and of miR-17-5p with Beclin1, as well. The binding site and the mutated sequence are shown in Fig. 4a. Beclin1 is an important part for formation of autophagosomes, and up-regulation of Beclin1 could activate autophagy [18]. To verify the possible role of HOTAIR, miR-17-5p and Beclin1 in cell autophagy, the expressions of miR-17-5p and Beclin1 in 786-O group, ACHN group, 786-O-R group, ACHN-R group, 786-O-R + sh-HOTAIR group, and ACHN-R + sh-HOTAIR group were determined. Then the dual-luciferase reporter gene assay and RIP assay were adopted to validate the binding of HOTAIR, miR-17-5p, and Beclin1. The findings displayed that miR-17-5p was lowly expressed in 786-O-R and ACHN-R cells in comparison with those in 786-O and ACHN cells, and knockdown of HOTAIR could restore the expressions of miR-17-5p in 786-O-R and ACHN-R cells (Fig. 4b) (*P* < 0.05). The expression pattern of Beclin1 was totally opposite to that of miR-17-5p in 786-O-R and ACHN-R cells (Fig. 4c, d) (*P* < 0.05).

The dual-luciferase reporter gene assay results manifested that in the wt-HOTAIR and wt-Beclin1 groups, the luciferase activity of miR-17-5p mimic was stronger than the luciferase activity of mimic NC (*P* < 0.05), and no obvious difference was revealed in the mut-HOTAIR and mut-Beclin1 groups (Fig. 4e, f).

Ago2 is a protein necessary for the binding of miRNA to its target gene, and in this study, the antibody of Ago2 was used for RIP assay to detect the binding of the miR-17-5p to its target gene. RIP assay showed highly expressed HOTAIR and Beclin1 in the Ago2 group as well as lowly expressed HOTAIR and Beclin1 in IgG group (Fig. 4g) (*P* < 0.01). The above findings revealed that HOTAIR serves as a ceRNA to mediate Beclin1 by competitively binding to miR-17-5p.

### MiR-17-5p decreases autophagy and sunitinib resistance of RC cells

**Fig. 5:**
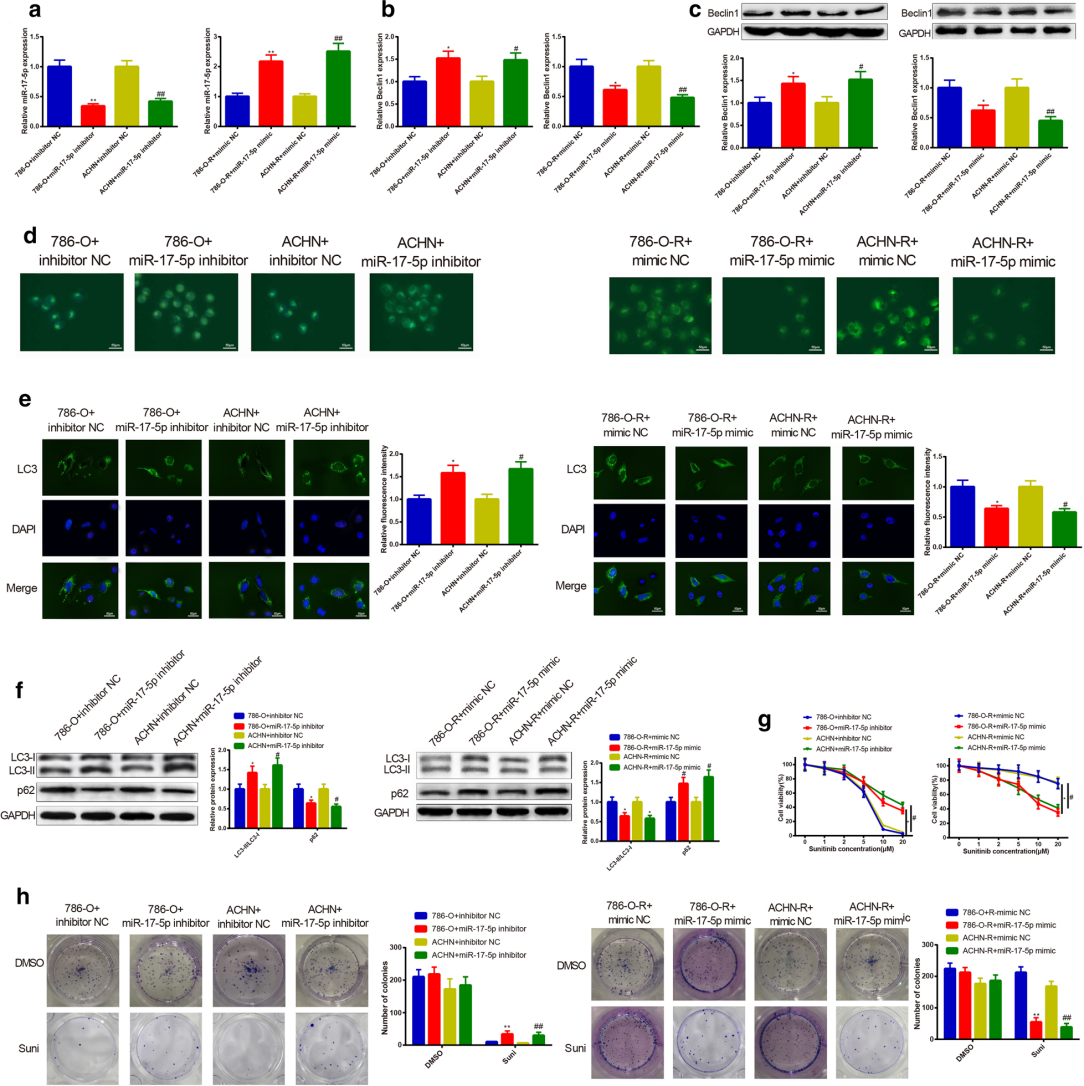
miR-17-5p inhibits autophagy and sunitinib resistance in RC cells. miR-17-5p gain and loss of function was performed in RC cells, in which parental cells were transfected with miR-17-5p inhibitor, and sunitinib-resistant cells were transfected with miR-17-5p mimic. After cell transfection, the mRNA expression of miR-17-5p was assayed by qRT-PCR (**a**). The mRNA and protein expressions of Beclin1 (**b**, **c**), LC3-II/LC3-I (**f**), and p62 (**f**) were tested by Western blot and qRT-PCR. Then miR-17-5p was found to negatively mediate Beclin1. MDC staining observed more formation of autophagic vacuoles in parental cells transfected with miR-17-5p inhibitor. Overexpression of miR-17-5p lessened the number of autophagic vacuole formation of sunitinib-resistant cells (**d**). After transfection, immunofluorescence revealed increased LC3 in parental cells and diminished expression intensity of LC3 in sunitinib-resistant cells (**e**). CCK-8 assay detection on the sensitivity of RC cells to sunitinib unfolded that parental cells transfected with miR-17-5p inhibitor had improved sunitinib resistance, while sunitinib-resistant cells transfected with miR-17-5p mimic possessed reduced sunitinib resistance of RC cells (**g**). The colony formation on cell colony formation ability exhibited that knockdown of miR-17-5p in 786-O or ACHN cells elevated clone number, whereas overexpression of miR-17-5p in 786-O-R or ACHN-R cells declined clone number (**h**); ^*^P < 0.05, ^**^P < 0.01, compared to 786-O + inhibitor NC or 786-O-R + mimic NC group; ^#^*P* < 0.05, ^##^*P* < 0.01, compared to ACHN + inhibitor NC or ACHN-R + mimic NC group; RC, renal cancer

Beclin1 is one of the well-proven regulators of cell autophagy [18]. Hence, we mainly focused on examining the role of miR-17-5p in autophagy and sunitinib resistance of RC cells. qRT-PCR and Western blot stated that transfection of miR-17-5p inhibitor could suppress miR-17-5p expression and elevate Beclin1 expression in both 786-O and ACHN cells, while transfection of miR-17-5p mimic in 786-O-R and ACHN-R cells results in elevated miR-17-5p expression and decreased Beclin1 expression (Fig. 5a–c) (*P* < 0.05). Those observations indicated the good transfection efficiency of miR-17-5p inhibitor and miR-17-5p mimic.

The effect of miR-17-5p inhibition on cell autophagy and sunitinib resistance was determined by MDC staining, immunofluorescence and Western blot. The experimental results stated that 786-O and ACHN cells transfected with miR-17-5p inhibitor had increased autophagic vacuoles (Fig. 5d), enhanced fluorescence intensity of LC3 (Fig. 5e), elevated LC3-II/LC3-I ratio (Fig. 5f), and improved cell clone formation ability (Fig. 5h) as well as repressed p62 expression (Fig. 5f) and suppressed cell sensitivity to sunitinib (Fig. 5g) (*P* < 0.05). These data indicated that knockdown of miR-17-5p could enhance cell autophagy and sunitinib resistance in RC.

To further prove the promotive effect of miR-17-5p knockdown on cell autophagy and sunitinib resistance, 786-O-R and ACHN-R cells were transfected with miR-17-5p mimic. The observations in 786-O-R and ACHN-R cells transfected with miR-17-5p mimic were totally opposite to findings obtained from 786-O and ACHN cells transfected with miR-17-5p inhibitor (Fig. 5a–h). Collectively, overexpression of miR-17-5p could suppress autophagy and sunitinib resistance of RC cells.

### Knockdown of HOTAIR reduces sunitinib resistance of RC cells in vivo

**Fig. 6:**
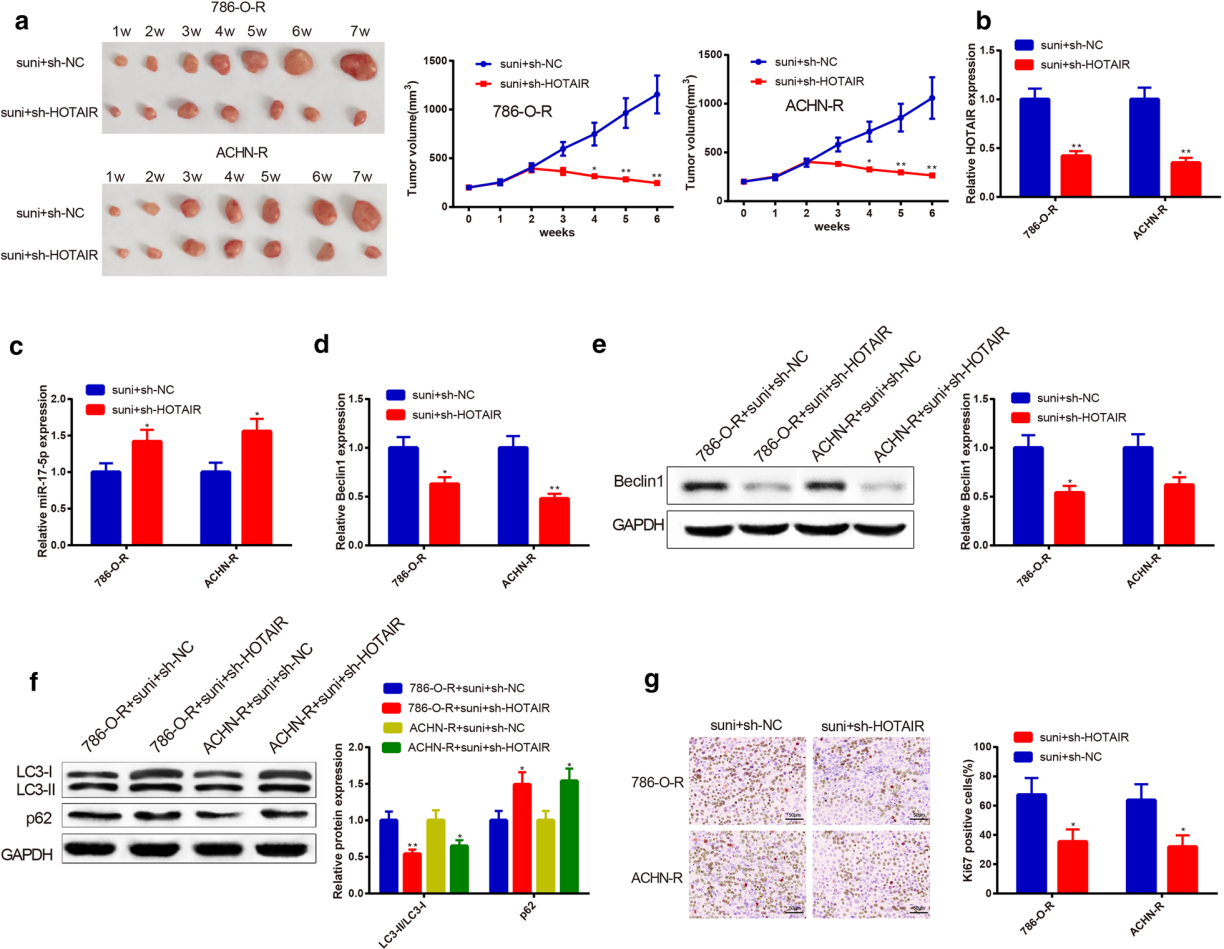
Effect of HOTAIR on sunitinib resistance of nude mouse xenografts. The nude mice were injected with 786-O-R or ACHN-R cells to establish xenograft models, followed by sunitinib treatment and sh-HOTAIR (sh-NC) injection. Tumor size was weekly recorded. Injection with sh-HOTAIR amplified the inhibitory effect of sunitinib on RC (**a**). qRT-PCR, Western blot, and immunohistochemistry were adopted to assay the expressions of HOTAIR (**b**), miR-17-5p (**c**), Beclin1 (**d**, **e**), LC3-II/LC3-I (**f**), p62 (**f**), and Ki67 (**g**). Injection with sh-HOTAIR heightened levels of miR-17-5p and p62, restrained HOTAIR expression, as well as decreased Beclin1 and LC3-II/LC3-I. Additionally, knockdown of HOTAIR in RC nude mice inhibited proliferation of tumor; ^*^P < 0.05, ^**^P < 0.01, compared to suni + sh-NC group or 786-O-R + suni + sh-NC or ACHN-R + suni + sh-NC; RC, renal cancer

To verify the effect of HOTAIR on sunitinib resistance in RC cells in vivo, luciferase-labeled 786-O-R and ACHN-R cells were injected into nude mice for construction of xenograft models. Noticeably, the suni + sh-HOTAIR group had decreased tumor size (Fig. 6a), reduced HOTAIR (Fig. 6b), LC3-II/LC3-I ratio (Fig. 6c), and Ki67 positive rate (Fig. 6d) as well as increased p62 expression (Fig. 6c) in comparison to the suni + sh-NC group (*P* < 0.05), illustrating that knockdown of HOTAIR in vivo could reduce sunitinib resistance in RC cells.

## Discussion

Sunitinib is the first-line treatment standard of care in advanced RC, while patients inevitably develop resistance to this drug [19, 20]. Furthermore, patients with metastatic RC often undergo antiangiogenic therapy resistance [21]. Hence, understanding the molecular mechanisms of intrinsic and acquired resistance to sunitinib may provide clues on how to circumvent this clinical problem. In the current study, we discovered that lncRNA HOTAIR could enhance RC cell autophagy and increase sunitinib resistance in RC cells. Thus, HOTAIR could act as a ceRNA to compete with Beclin1 to bind miR-17-5p, and therefore regulate cell autophagy and decrease sensitivity to sunitinib in RC cells.

Autophagy, an intra-cellular self-defense mechanism can be used by tumor cells as a defense apoptotic cell death, which enhance the resistance of tumor cells to chemotherapeutic agents while inhibition of autophagy is reported to sensitize lung cancer cells to chemotherapy [18] To explore the possible mechanism of cell autophagy on sunitinib resistance in RC cells, RAP and 3-MA were used to activate or inactivate cell autophagy in parental cells or sunitinib-resistant cells. The results highlighted that RAP treatment increased autophagic vacuoles, enhanced expressions of LC-3, LC3-II/LC3-I, enhanced colony formation ability, reduced cell sensitivity toward sunitinib and diminished p62 level in parental cells. Additionally, suppressed autophagy and sunitinib resistance of RC cells were obtained with the application of 3-MA to 786-O-R and ACHN-R cells. Previous research supported our findings that the repression of autophagic flux and sequestration in lysosomes may herald resistance to sunitinib in renal clear cell carcinoma [8]. These results indicated that cell autophagy confers an indispensable role in RC cell resistance to sunitinib, and autophagy suppression may enhance RC cell sensitivity to sunitinib.

LncRNAs and ncRNAs have been implicated in kinds of cancers as important biomarkers [22, 23]. LncRNA HOTAIR aggravates the malignancy of renal cell carcinoma and is associated with the poor prognosis of many cancers [24]. In this study, we initially found that sunitinib-resistant RC cells had a higher HOTAIR expression than parental cells. This finding prompted us to probe whether HOTAIR elicits a regulatory effect on the sunitinib resistance of RC cells. To this end, loss-of-function HOTAIR was achieved in 786-O-R and ACHN-R cells. Interestingly, we found that knockdown of HOTAIR can inhibit cell autophagy and drug resistance in RC cells, indicating the implication of HOTAIR in sunitinib resistance. Similarly, a former study of Mengyao Sun et al. reported that HOTAIR-mediated autophagy influences the cisplatin-induced resistance in endometrial cancer cells [25]. Moreover, Xia Wang et al. highlighted that silencing of HOTAIR impeded autophagy and accelerated apoptosis to reduce oral squamous cell carcinoma resistance to cisplatin [26]. Another example proposed that the knockdown of HOTAIR inhibits ULK1 phosphorylation-mediated autophagy to refrain drug resistance of non-small cell lung cancer cells [27]. In general, our findings stated that the knockdown of HOTAIR improved the sensitivity of RC cells to sunitinib. However, it is uncertain how HOTAIR might be responsible for autophagy-related sunitinib resistance.

The downregulation of miR-17-5p expression caused Belin1 upregulation may participate in paclitaxel resistance of lung cancer [18]. Considering the biological function of miR-17-5p, we next sought to explore the effect of miR-17-5p on sunitinib resistance of RC cells. MDC, immunofluorescence and Western blot were utilized and the collected observation found that miR-17-5p silencing in parental cells lead to enhanced sunitinib resistance and increased cell autophagy ability, while overexpression miR-17-5p in sunitinib-resistant cells potentiated RC cell sensitive to sunitinib and restrained cell autophagy. Hence, miR-17-5p may raise the sensitivity of RC cells to sunitinib. Additionally, the dual-luciferase reporter gene and RIP assay in this study presented the binding relationships between HOTAIR, miR-17-5p and Beclin1. In this regard, it is reasonable to conjecture that HOTAIR can down-regulate the expression of miR-17-5p to enhance the expression of Beclin1 in RC cells, thus promoting cell autophagy. Beclin1, an autophagy-related factor, has significantly up-regulated expression when autophagy occurs [28]. Overexpression of miR-17-5p in 786-O-R and ACHN-R was found to inhibit Beclin1-mediated cell autophagy. Previous researches supported our finding that miR-17-5p inhibits Beclin1-mediated autophagy to heighten the radiosensitivity of glioma cells [29]. MiRNAs and lncRNAs are important ncRNAs, which have long been explored for their roles as diagnostic and prognostic biomarkers of cancers as well as regulators of tumorigenesis [9]. In gallbladder cancer, lncRNA MALAT1 has been documented to function as a ceRNA to negatively modulate MCL-1 expression by sponging miR-363-3p [30]. Moreover, HOTAIR has been introduced to function as a ceRNA for miR-217 to facilitate RC progression partly via HIF-1α/AXL signaling [31]. Consistently, in vivo experiment also showed that injection with sh-HOTAIR and sunitinib reduced tumor size and inhibited expressions of autophagy-related proteins. Collectively, HOTAIR can negatively target miR-17-5p to regulate Beclin1 mediated cell autophagy in RC cells.

In summary, the down-regulation of HOTAIR could enhance the sensitivity of RC cells to sunitinib through up-regulating miR-17-5p to suppress Beclin1-mediated cell autophagy. Therefore, this study suggested that the possibility of HOTAIR serving as a potential candidate for restoring sunitinib sensitivity in the chemotherapy of RC. Nonetheless, these results must be interpreted with caution and more studies are required to validate the results of the current study. Furthermore, it is also important to further explore the possible mechanism of cell autophagy in regulating sunitinib resistance in RC cells, which will enable us to have a comprehensive and better understanding on restoring sunitinib sensitivity in RC cells.

## Data Availability

The datasets used or analyzed during the current study are available from the corresponding author on reasonable request.

## Abbreviations

LncRNA: Long non-coding RNA
RC: Renal cancer
RCC: Renal cell carcinoma
TKIs: Tyrosine kinase inhibitors
EMT: Mesenchymal transformation
FBS: Fetal bovine serum
qRT-PCR: Quantitative reverse transcription polymerase chain reaction
MDC: Monodansylcadaverine
RIP: RNA immunoprecipitation
SD: Co-standard deviation
ANOVA: One-way analysis of variance
ncRNAs: Noncoding RNAs
